# Functional Genomic Analyses of Exopolysaccharide-Producing *Streptococcus thermophilus* ASCC 1275 in Response to Milk Fermentation Conditions

**DOI:** 10.3389/fmicb.2019.01975

**Published:** 2019-08-23

**Authors:** Qinglong Wu, Hung Chu, Aparna Padmanabhan, Nagendra P. Shah

**Affiliations:** ^1^School of Biological Sciences, The University of Hong Kong, Pokfulam, Hong Kong; ^2^Texas Children’s Microbiome Center, Department of Pathology, Texas Children’s Hospital, Houston, TX, United States; ^3^Department of Pathology and Immunology, Baylor College of Medicine, Houston, TX, United States

**Keywords:** exopolysaccharide, *Streptococcus thermophilus*, pH, proteome, transcriptome

## Abstract

Exopolysaccharide (EPS) produced from dairy bacteria improves texture and functionalities of fermented dairy foods. Our previous study showed improved EPS production from *Streptococcus thermophilus* ASCC1275 (ST1275) by simple alteration of fermentation conditions such as pH decrease (pH 6.5 → pH 5.5), temperature increase (37°C → 40°C) and/or whey protein isolate (WPI) supplementation. The iTRAQ-based proteomics in combination with transcriptomics were applied to understand cellular protein expression in ST1275 in response to above shifts during milk fermentation. The pH decrease induced the most differentially expressed proteins (DEPs) that are involved in cellular metabolic responses including glutamate catabolism, arginine biosynthesis, cysteine catabolism, purine metabolism, lactose uptake, and fatty acid biosynthesis. Temperature increase and WPI supplementation did not induce much changes in global protein express profiles of ST1275 between comparisons of pH 5.5 conditions. Comparative proteomic analyses from pairwise comparisons demonstrated enhanced glutamate catabolism and purine metabolism under pH 5.5 conditions (Cd2, Cd3, and Cd4) compared to that of pH 6.5 condition (Cd1). Concordance analysis for differential expressed genes (DEGs) and DEPs highlighted down-regulated glutamate catabolism and up-regulated arginine biosynthesis in pH 5.5 conditions. Down regulation of glutamate catabolism was also confirmed by pathway enrichment analysis. Down-regulation of EpsB involved in EPS assembly was observed at both mRNA and protein level in pH 5.5 conditions compared to that in pH 6.5 condition. Medium pH decreased to mild acidic level induced cellular changes associated with glutamate catabolism, arginine biosynthesis and regulation of EPS assembly in ST1275.

## Introduction

Exopolysaccharide (EPS) produced by food-grade bacteria such as lactic acid bacteria (LAB) and bifidobacteria improves texture and functionality of fermented foods due to its stabilizing/texturizing properties as well as modulation of immune responses (Ruas-Madiedo et al., 2002; Welman and Maddox, 2003; Galle and Arendt, 2014; Hidalgo-Cantabrana et al., 2014). Thus, many studies have been carried out on the isolation of EPS producers, optimization of EPS production, and chemical and functional characterization of EPS (Ruas-Madiedo and de los Reyes-Gavilán, 2005; Caggianiello et al., 2016). EPSs from dairy starters not only show well-documented functionalities mentioned above, but also are fermentable substrates termed as “prebiotics” for modulating human gut microbiome (Badel et al., 2011; Caggianiello et al., 2016; Salazar et al., 2016). Many studies have adopted non-milk-based media such as de Man, Rogosa and Sharpe (MRS) broth for optimizing EPS production from LAB (Kimmel et al., 1998; Aslim et al., 2005; Desai et al., 2006; Vera Pingitore et al., 2016), however, these optimized conditions may not be applied to bacterial EPS production in milk-based media because many strains of LAB lack extracellular proteolytic activity that is crucial to bacterial survival in milk environment (Buckenhüskes, 1993). However, the cell envelope protease (PrtS) attributed to proteolysis is well distributed in *Streptococcus thermophilus* of dairy origin (Delorme et al., 2010). Therefore, high EPS-producing dairy *Str. thermophilus* has become a promising source to make EPS-enriched fermented milks (Iyer et al., 2010).

Several studies have demonstrated high EPS production from non-starter LAB (NSLAB) such as the *Lactobacillus casei* group, *Lb. acidophilus*, *Lb. helveticus*, *Lb. brevis*, and *Lb. plantarum* (Welman and Maddox, 2003; Caggianiello et al., 2016). For example, *Lb. rhamnosus* RW-9595M produced the highest amount of EPS in a chemically defined medium among the reported strains of LAB and bifidobacteria (Bergmaier et al., 2005). Although NSLAB strains have been reported to improve the quality of some fermented dairy foods (Leroy and De Vuyst, 2004; Settanni and Moschetti, 2010), those NSLAB strains could be potentially introduced as adjunct starters considering their weak proteolytic activities and low acidifying rates (Buckenhüskes, 1993; Sasaki et al., 1995). Thus, numerous strains of typical dairy starters including *Str. thermophilus*, *Lb. delbrueckii* subsp. *bulgaricus* (*Lb. bulgaricus*) and *Lactococcus lactis* subsp. *lactis* (*Lc. lactis*) have been characterized for EPS production but their yields of EPS were not enough high to improve the functionalities of the fermented milk product. This may limit their usage for dairy products where EPS production is required (Frengova et al., 2000). Among them, *Str. thermophilus* ASCC 1275 (ST1275), a conventional dairy starter, has been identified in our previous study as a high EPS producer in milk, and its EPS production could be simply improved by adjusting the fermentation conditions such as pH, temperature or supplementing milk with limited amount of whey protein isolate (WPI), a by-product from the cheese-making (Zisu and Shah, 2003). Characteristics of EPS from ST1275 have been investigated intensively in our lab for use in fermented milk products (Amatayakul et al., 2006a, b; Purwandari et al., 2007; Li and Shah, 2014, 2016).

**FIGURE 1:**
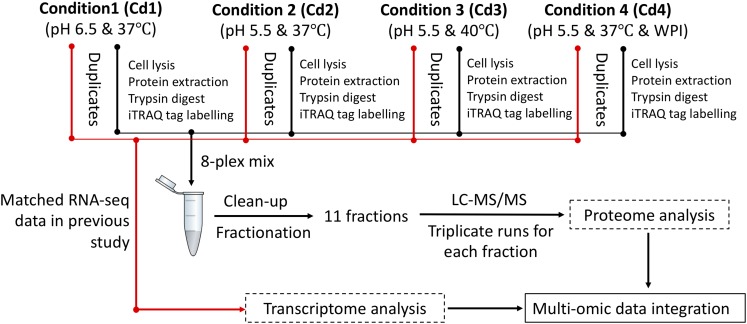
Experimental design for iTRAQ-based proteomic and matched transcriptome analysis on ST1275 under four milk fermentations. Duplicate batches of milk fermentation were carried out for each condition.

We previously optimized milk fermentations for improving EPS biosynthesis in ST1275. Specifically, we focused on four types of milk fermentations for comparisons in that study: condition 1 (Cd1) – pH 6.5 and 37°C; condition 2 (Cd2) – pH 5.5 and 37°C; condition 3 (Cd3) – pH 5.5 and 40°C; condition 4 (Cd4) – pH 5.5 and 37°C with 0.5% (wt/vol) WPI supplementation to the reconstituted skim milk (RSM) (Zisu and Shah, 2003); there were slight changes in the bacterial growth of ST1275 cultivated in milk under pH 5.5 conditions (Cd2, Cd3, and Cd4) compared to Cd1, but a major increase in EPS production from this organism was observed (Zisu and Shah, 2003). This motivated us to understand physiological changes in regards to EPS biosynthesis in ST1275. Moreover, ST1275 produced the highest amount of EPS in milk among the reported strains of *Str. thermophilus* (Wu et al., 2014). Recently, our genomic study provided detailed insights into global gene annotation for ST1275 thus making transcriptomic and proteomic analysis possible due to the viability of an accurate database for nucleotide alignment and peptide-spectrum match scoring, respectively. In addition, our recent preliminary transcriptome study for ST1275 identified differentially expressed genes (DEGs) under optimized fermentation conditions (Cd2, Cd3, and Cd4) when compared to the pH 6.5 condition (Cd1) (Wu and Shah, 2018). Due to the importance of *Str. thermophilus* in dairy industry (Iyer et al., 2010), ST1275 was used as a model high EPS-producing strain in this study to understand the role of environmental conditions in shaping its protein expression. Early two-dimensional electrophoresis approach could identify a narrow spectrum of differential expressed proteins, for example, pyruvate formate-lyase was recognized as a formate supplier for anabolic purposes in *Str. thermophilus* LMG18311 (Derzelle et al., 2005). More precise shotgun proteomics approach based on nano-LC/MS/MS was utilized to reveal the bacteriophage-induced clustered regularly interspaced short palindromic repeats (CRISPR)-associated (Cas) proteins for protecting phage invasion in *Str. thermophilus* DGCC7710 (Young et al., 2012). In the present study, isobaric tags for relative and absolute quantitation (iTRAQ)-based proteomics, with the inclusion of fractionation step, could provide high resolution to proteome profiling (Latosinska et al., 2015). In this study, we applied the iTRAQ-based proteomics approach (Figure 1) to detect the protein expression of ST1275 in response to pH 5.5 conditions; further paired analysis of differentially expressed proteins (DEPs) based on proteome data and DEGs based on our previous transcriptome data (Wu and Shah, 2018) for matched samples was also performed.

## Materials and Methods

### Bacterial Cultivation and Batch Milk Fermentation

*Streptococcus thermophilus* ASCC 1275 (ST1275) was cultivated in Difco^TM^ M17 broth (BD Company, Franklin Lakes, NJ, United States) containing 1% (w/v) lactose at 37°C for 18 h prior to the inoculation into 10% (w/v) RSM (initial density was about 10^6^ CFU/mL). The RSM medium used for batch fermentation was prepared from Nestlé^®^ Carnation^®^ skimmed milk powder (Nestlé Hong Kong Ltd., Kowloon, Hong Kong) consisting of 27 g protein, 42 g lactose and 9 g of maltodextrin and gluco-oligosaccharide per 100 g of the powder. A portion of 0.5% (wt/vol) WPI (Nature’s Best^®^, Hauppauge, NY, United States) containing 52% protein, 20% chloride, 7% sodium, 4% calcium, 4% iron, 2% magnesium, and 2% phosphorous was supplemented to the RSM before autoclaving if specified. RSM was autoclaved at 120°C for 15 min.

Milk batch fermentations under different pH, temperature and WPI were followed as in our previous study for this strain (Zisu and Shah, 2003). Cultures were centrifuged to remove supernatant and were re-suspended in the same volume of sterile RSM for inoculation. Milk fermentation was carried out in an assembled bioreactor – GLS 80^®^ stirred reactor (DURAN Group, Mainz, Germany). The pH probe was sterilized by 1 N NaOH solution and was then inserted into the bioreactor through one of the ports in screw cap. Another two ports were connected to 6 M NaOH and 50% (wt/vol) citric acid solutions which were used for adjusting the pH of milk. The milk fermentations from four conditions (Cd1, Cd2, Cd3, and Cd4) were stopped after 6 h since the cells were in late log-growth phase (data not shown) because ST1275 is a highly milk-adapted strain. Such time point would be reasonable for comparisons between conditions since EPS production rate is high during stationary phase. About 10 mL of fermented milk after sampling was immediately treated with 3.3 mL of 1 M trisodium citrate (Derzelle et al., 2005); bacterial cell pellet was harvested by centrifugation and were frozen immediately with liquid nitrogen and were stored at −80°C for protein extraction. Experimental procedure for peptide library construction and proteomic analysis is outlined in Figure 1. All the samples used for proteomic analysis also have transcriptome data from the same batch fermentation at the same time point (6 h). All the transcriptome analysis has been published in our previous study (Wu and Shah, 2018).

### Protein Extraction

Bacterial cell pellets kept at −80°C were thawed on ice and were washed twice with ice-cold 200 mM sodium phosphate buffer (pH 6.4) and were re-suspended in lysis buffer containing 150 mM NaCl, 50 mM Tris (pH 8.4), 0.1% (w/v) sodium dodecyl sulfate (SDS), 2 mM β-mercaptoethanol and Roche cOmplete^TM^ Mini Protease Inhibitor Cocktail [1 tablet containing ethylenediaminetetraacetic acid (EDTA) yielding 1 mM EDTA in 10 mL solution]. Disruption of bacterial cells was carried out by a cell lysis probe sonicator (Bioland Instrument, Xi’an, China) with the operating frequency of 20 kHz and the ultrasonic power of 100 W. The disruption was operated for 3 s with 2 s intervals between runs for a total duration of 5 min in an ice bath. Supernatant containing proteins was collected after centrifugation at 12,000 *g* for 15 min at 4°C. Protein concentration was measured by Bradford Reagent (Sigma-Aldrich); bovine serum albumin (BSA) was dissolved in the same lysis buffer to make protein standards for generating standard curve.

### iTRAQ-Based Peptide Labeling

An aliquot of 100 μg of extracted proteins per sample was used in the shotgun proteomic analysis. Acetone precipitation of proteins, protein disulfides reduction, cysteine blocking and trypsin-based digestion, and iTRAQ tag labeling were carried out with iTRAQ^®^ Reagents 8-plex Kit (AB Sciex, Framingham, MA, United States) as per manufacturer’s instructions (Liang et al., 2016).

### LC/MS/MS Analysis

All the tagged peptides were combined together to proceed with the desalting process and fractionation in Agilent high-performance liquid chromatography (LC) equipped with a C18 column; the buffer and solvent (acetone) in the collected fractions were removed by using C18 SepPak purification kit (Waters, Milford, MA, United States); nano-LC/MS/MS analysis of purified peptides (a total number of 11 fractions) was carried out in TripleTOF 5600 system fitted with a Nanospray III source (AB Sciex) as per previously described (Lau et al., 2011). To increase the detection limitation of MS, we performed three technical repeated runs for LC/MS/MS analysis on each fraction.

### Proteomic Data Analysis and Statistical Testing

Downstream analysis of MS data was carried out with ProteinPilot software version 5.0.1.0. Since ST1275 has been completely sequenced (Wu et al., 2014), a decoy database of reverse translated sequences of ST1275 genome was generated for performing target-decoy database search. Mass spectrums (MS2) were searched by using Paragon algorithm against the built target-decoy database. Proteomics System Performance Evaluation Pipeline Software (PSPEP), an add-on function in ProteinPilot software) was used to perform both global and local FDR analysis on Paragon search results. protein hits from decoy database (labeled with “REVERSED” in the header identifier of protein sequences) were manually removed from the result output. A minimal unused ProtScore (corresponding to 1% critical FDR) with at least two peptides, which were identified with a confidence level of above 95%, was used as threshold for filtering protein list. Since each condition has duplicate samples and each trypsin-digested peptide library was labeled with one iTRAQ tag, all the four replicated ratios (termed as “fold change”), which were generated from the comparison between two conditions, were used for statistical analysis by using one sample t-test based on the equation: $t=\frac{m-\mu \;}{s/\sqrt{n-1\,}\;}$ (where m is sample mean, n is sample size, s is sample standard deviation, μ is the theoretical value) in Prism GraphPad version 5.04 (Lau et al., 2011; Liang et al., 2016). In this study, two theoretical values: 1.30 (1.30-fold up-change) and 0.7692 (1.30-fold down-change) were used for identifying DEPs with *p* < 0.05 (two-tailed). Another theoretical value set to 1 was used to generate volcano plot based on log2 (median fold-change) against −log10 (*p*-value) for all the comparisons between two conditions.

### Transcriptome Analysis for Matched RNA-Seq Data

We followed the same analysis procedure for the comparisons (Cd2 vs. Cd1, Cd3 vs. Cd1, Cd4 vs. Cd1, Cd3 vs. Cd2, and Cd4 vs. Cd2) of matched transcriptome data based on our previous study (Wu and Shah, 2018). Briefly, Illumina paired-end sequencing reads (100 bases per read) after quality filtering and trimming were aligned to ST1275 genome by Tophat package (v2.1.1) incorporating Bowtie v2.1.0; HTSeq package (v0.9.1) was used to generate read counts per gene for each sample for the aligned output generated in previous alignment step; the edgeR’s quasi-likelihood pipeline incorporating the trimmed mean of M-values (TMM) normalization, quasi-likelihood F-test and FDR analysis was used for downstream analysis of read count table to generating the DEGs. The only difference between our previous RNA-seq analysis and this study is the number of replicates: duplicates per group used in the present study but triplicated samples were used in the previous study (Wu and Shah, 2018).

### Functional Classification for Each Coding Sequence

Clusters of Orthologous Groups (COG) annotation and KEGG Orthology (KO) functional assignment for coding sequences of ST1275 were downloaded from Integrated Microbial Genomes and Microbiomes (IMG/M) database. Another functional annotation of coding sequences of ST1275 was carried out with PANTHER classification system by using PANTHER’s hidden Markov models (HMM) Scoring tool – pantherScore2.1.pl with PANTHER HMM library version 13.1 (Mi et al., 2013, 2017).

### Multi-Omics Data Integration for Paired Transcriptome and Proteome Results

DEPs from proteome analysis and DEGs from transcriptome analysis from 5 comparisons (Cd2 vs. Cd1, Cd3 vs. Cd1, Cd4 vs. Cd1, Cd3 vs. Cd2, and Cd4 vs. Cd2) were performed with concordance analysis, which searched common targets (protein-coding genes) shared by at least two comparisons. The PANTHER accession numbers for protein-coding genes (DEPs/DEGs) together with fold changes from either proteome analysis or transcriptome analysis were used for pathway enrichment analysis in PANTHER website.

## Results

### Summary of Transcriptome Analysis and iTRAQ-Based Proteomic Analysis

Due to unavailability of the same skim milk powder that we used in the previous study (Zisu and Shah, 2003), we used a fortified skim milk containing extra maltodextrin and gluco-oligosaccharides (Glc-OS) for milk fermentation. Based on KEGG metabolism pathways for ST1275, gene T303_08530 encoding a cytoplasmic α-amylase has the potential for hydrolyzing intracellular maltodextrin and Glc-OS into maltose which could be further catabolized into glucose. However, our bacterial growth assay demonstrated that maltose and starch were not able to stimulate the growth of ST1275 in the M17-based media which is suitable and rich medium for *Str. thermophilus* (Supplementary Figure S1). This suggests that the membrane uptake for both maltose and starch or other glycogen including maltodextrin and Glc-OS may not happen in ST1275. Thus, we believe that the extra polysaccharide in the RSM would have limited effect on protein expression of ST1275.

**FIGURE 2:**
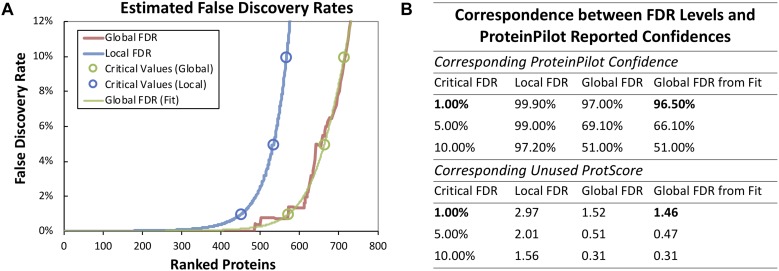
Summary of proteomic analysis with ProteinPilot software. **(A)** False discovery rate analysis based on the target-decoy database search strategy. **(B)** FDR levels and ProteinPilot reported confidences. A critical FDR with 1% which corresponding to Unused Protscore of 1.46 from global FDR (fit) was used for filtering protein.

Based on our previous genome annotation for ST1275, there are 1771 coding sequences in its genome (Wu et al., 2014), mRNA-seq approach detected 1702 transcripts (Supplementary Table S1), whereas 794 proteins (data not shown) were detected from 33 runs (triplicate runs for each peptide fraction) in our fractionated iTRAQ-based proteomic workflow. However, the 536 proteins (67.3% of total detected proteins) passed two critical analysis thresholds: (1) the unused ProtScore for each protein was higher than 1.46 (corresponding to critical false discovery rate (FDR) of 1%), and (2) at least 2 unique peptides (95% confidence) were detected from the same protein source (Supplementary Table S2 and Figure 2). Although the fractionation of iTRAQ-labeled peptides (from trypsin-digested proteins) has been shown to improve the resolution of mass spectrometry (MS)-based proteomics (Latosinska et al., 2015), low abundant proteins extracted from complex matrix still may not be detected by this method. However, detected proteins which passed the analysis threshold mentioned above would be able to provide confident insights into the protein expression in ST1275 under four fermentation conditions.

Based on 536 protein-coding genes detected by both transcriptome and proteome, correlation analyses were carried out for those common shared targets as shown in Supplementary Figure S2. We observed low Pearson’s *r* values (correlation coefficient) ranging from almost 0 to 0.36 for all comparisons. Specifically, slightly higher positive correlation was observed in three comparisons (Cd2 vs. Cd1, *r* = 0.332; Cd3 vs. Cd1, *r* = 0.361; Cd4 vs. Cd1, *r* = 0.305) due to pH decrease than those of other two comparisons (Cd3 vs. Cd2, *r* = 0.09; Cd4 vs. Cd2, *r* = 0.007) focusing on temperature increase and WPI supplementation. This discordance between transcriptome and proteome has been commonly observed in previous studies; this may be related to post-transcription machinery (Haider and Pal, 2013). Therefore, we focused more on the integration of proteome data since the proteome is more functional than transcriptome in regards to cellular and functional shifts in ST1275 in response to environmental changes. However, mRNA-based transcriptome results have been validated by qPCR assay on both DEGs and non-DEGs in our previous study (Wu and Shah, 2018). Thus, transcriptome results for those matched samples could be used to validate or confirm proteomic findings, especially concordant DEGs and DEPs from the same comparison between two conditions.

### DEPs From Comparisons Between pH 6.5 Condition (Cd1) and pH 5.5 Conditions (Cd2, Cd3, or Cd4)

**FIGURE 3:**
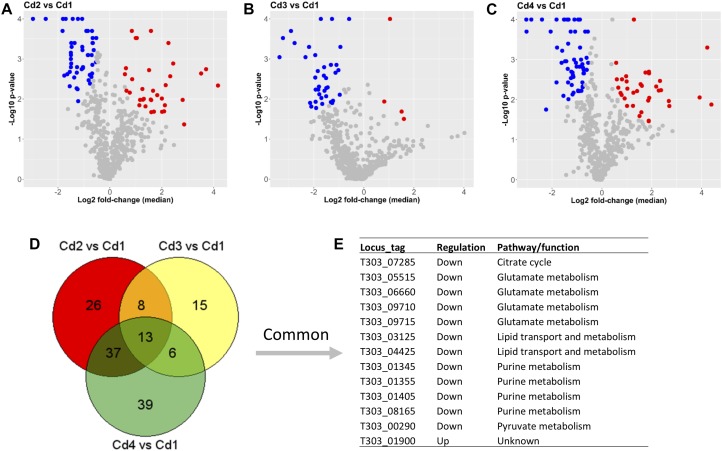
Comparisons between optimized conditions (Cd2, Cd3, and Cd4) and pH 6.5 condition (Cd1) based on proteome analysis. **(A)** DEPs from the comparison (Cd2 vs. Cd1). **(B)** DEPs from the comparison (Cd3 vs. Cd1). **(C)** DEPs from the comparison (Cd4 vs. Cd1). **(D,E)** Common DEPs shared by three comparisons. Dot (detected proteins) highlighted in blue indicates down-DEPs, dot (detected proteins) highlighted in red indicates up-DEPs.

One of our main comparisons was carried out for pH decrease since EPS yields in Cd2, Cd3, and Cd4 were much higher than that in Cd1 as suggested in our previous study (Zisu and Shah, 2003). In the present study, proteins with at least 1.3-fold up- or down-change (*p* < 0.05) are considered as DEPs. Lists of DEPs from the three comparisons (Cd2 vs. Cd1, Cd3 vs. Cd1, and Cd4 vs. Cd1) are showed in Supplementary Table S2. As also shown in Figures 3A–C, there were 49 down-DEPs and 35 up-DEPs in Cd2 due to pH decrease (from pH 6.5 to 5.5) when compared to Cd1; similarly, 60 down-DEPs and 35 up-DEPs were observed from the comparison – Cd4 vs. Cd1, those DEPs were induced by both pH decrease (from pH 6.5 to pH 5.5) and WPI supplementation; however, there were only 4 up-DEPs in Cd3 because of pH decrease (from pH 6.5 to pH 5.5) and temperature increase (from 37 to 40°C) when compared to Cd1 though there were 38 down-DEPs observed. When those DEPs from the above three comparisons were compared to each other, 13 concordant DEPs including 12 down-DEPs and 1 up-DEPs were found to be associated with several pathways such as glutamate metabolism, purine biosynthesis, and lipid transport and metabolism (Figures 3D,E). Since those down-regulated pathways in pH 5.5 condition were not linked to acidic resistance, they might be associated with cellular function. Gene (T303_01900)-encoded hypothetical protein (73 amino acids) with 5 unique peptides identified was almost 2-fold up-regulated in all three comparisons (Supplementary Table S2). Structural modeling for this protein (encoded by T303_01900) by Protein Homology/analogY Recognition Engine V 2.0 (PHYRE2) demonstrated that all residues have been modeled with 100% confidence based on the template 4IAJ from Protein Data Bank (PDB), a conserved domain protein (gene locus ID: SP_1775) from *Streptococcus pneumoniae* TIGR4; however, further characterization is still necessary to understand its function and role under acidic condition in ST1275.

As for EPS biosynthesis, only five proteins (T303_06336, T303_06385, T303_06400, T303_06410 and T303_06415) out of 20 EPS assembly associated proteins were confidently detected by iTRAQ-based method (Supplementary Table S2); only EpsB (T303_06415) was identified as the down-regulated DEP from two comparisons – Cd3 vs. Cd1 and Cd4 vs. Cd1, but not from the comparison between Cd2 and Cd1.

### Effects of pH Decrease (Cd2 vs. Cd1) on Stress Responses in ST1275

Unlike Cd3 vs. Cd1 and Cd4 vs. Cd1, comparison between Cd2 and Cd1 focused on pH decrease only. We observed major changes in expression of stress-induced proteins: (1) general stress-induced proteins (T303_04835, 4.77-fold up-regulated; T303_00885, 17.90-fold up-regulated; T303_07635, 4.43-fold up-regulated) showed responses to general stresses; the *dps* gene (T303_04835) has been demonstrated to contribute acid tolerance and oxidative stress tolerance in other organisms such as *Escherichia coli* and *Lactococcus lactis* (Choi et al., 2000; Budin-Verneuil et al., 2005); (2) alkaline shock proteins (T303_00870, 3.01-fold up-regulated; T303_00880, 11.42-fold up-regulated); (3) up-regulated proteins (T303_04825, 3.17-fold; T303_05985, 3.02-fold; T303_9840, 13.03-fold) and one down-regulated protein (T303_04430, 7.69-fold) showed potential against oxidative stresses. Since the pH level is the only parameter changed during milk fermentation under Cd1 and Cd2, it appears that cellular adaptation to mildly acidic condition (pH 5.5) induced cross-protection against oxidative stress. This mild pH-triggered oxidative stress response phenomenon has also been observed in *E. coli* (Maurer et al., 2005).

### Effects of pH Decrease (Cd2 vs. Cd1) on Amino Acid Metabolism in ST1275

Among the differentially expressed proteins due to pH decrease (Cd2 vs. Cd1), some were associated with arginine biosynthesis (Table 1). The intermediate metabolites – glutamate and pyruvate are the central precursors for arginine biosynthesis in ST1275. Typically, the conversion process of serine to pyruvate appears to be improved because of the up-regulated proteins (at least 4-fold) – T303_05420 and T303_05425 in regards to the pH 5.5 adaptation; serine has been documented as a mild essential amino acid for the growth of *Str. thermophilus* (Pastink et al., 2009). Pyruvate is an important precursor for many biological processes (Liu, 2003), however, we only observed the 2.7-fold down-regulation of ketol-acid reductoisomerase (T303_00290) that is involved in valine, leucine, and isoleucine biosynthesis. In addition to the down-regulation of glutamate dehydrogenase (T303_03260; 2.56-fold down-change) and glutamine synthetase (T303_09710; 2.38-fold down-change), it appears that more glutamate can be utilized for arginine biosynthesis in ST1275 under Cd2 condition because of down-regulated glutamine synthesis using glutamate (T303_09710); the *glnR* (T303_09715)-mediated repression of *glnR* operon (T303_09710 and T303_09715) was also observed in other streptococci under acidic shock (Chen et al., 2016).

**TABLE 1:** List of DEPs from the comparison – Cd2 vs. Cd1.

**Locus_tag**	**Fold change (median)**	**GenBank annotation**	**Note**
**Amino acid transport and metabolism**			
T303_00290	0.36	Ketol-acid reductoisomerase	Pyruvate metabolism
T303_05515	0.35	Glucosamine-fructose-6-phosphate aminotransferase	Glutamate metabolism
T303_06660	0.39	Glutamine ABC transporter permease	Glutamate metabolism
T303_09710	0.43	Glutamine synthetase	Glutamate metabolism
T303_09715	0.30	Repressor of the glutamine synthetase	Glutamate metabolism
T303_03455	0.48	Homoserine kinase	Threonine biosynthesis
T303_00325	1.54	Threonine synthase	Threonine metabolism
T303_03725	0.42	Carbamoyl phosphate synthase small subunit	Glutamate metabolism
T303_03260	0.39	Glutamate dehydrogenase	Glutamate metabolism
T303_03130	0.32	*S*-ribosylhomocysteinase	Methionine biosynthesis
T303_05130	0.35	5-methyltetrahydropter oyltriglutamate-homocysteine methyltransferase	Methionine biosynthesis
T303_07020	0.34	Phosphoribosyl-ATP pyrophosphatase	Histidine biosynthesis
T303_05425	7.26	Cystathionine beta-lyase	Cysteine catabolism
T303_05420	4.25	Cysteine synthase	Cysteine catabolism
T303_03425	4.00	*N*-acetyl-gamma-glutamyl-phosphate reductase	Arginine biosynthesis
T303_03440	3.54	Acetylornithine aminotransferase	Arginine biosynthesis
T303_03435	2.42	Acetylglutamate kinase	Arginine biosynthesis
T303_09105	4.34	Amino acid ABC transporter substrate-binding protein	
T303_08290	2.47	Amino acid ABC transporter substrate-binding protein	
T303_08720	1.81	Amino acid ABC transporter substrate-binding protein	
T303_04105	1.79	Branched-chain amino acid aminotransferase	
**Carbohydrate transport and metabolism**			
T303_04850	0.62	Glucokinase	
T303_02705	0.53	Transketolase	
T303_07870	0.49	Lactose/galactose permease	
T303_06080	5.42	Maltodextrin phosphorylase	
T303_06085	5.00	4-alpha-glucanotransferase	
**Stress response**			
T303_00885	17.90	Hypothetical protein	General stress response
T303_07635	4.43	Hypothetical protein	General stress response
T303_04835	4.77	DNA protection during starvation protein	Response to multiple stresses
T303_04430	0.13	Alkylhydroperoxidase	Response to oxidative stress
T303_04825	3.17	Superoxide dismutase	Response to oxidative stress
T303_05985	3.02	Lipid hydroperoxide peroxidase	Response to oxidative stress
T303_09840	13.03	Thioredoxin	Response to oxidative stress
T303_00870	3.01	Membrane protein	Response to base shock
T303_00880	11.42	General stress regulator, Gls24 family	Response to base shock
T303_09015	2.00	Universal stress protein UspA	Response to nutrient exhaustion
**Nucleotide transport and metabolism**			
T303_01335	0.36	Phosphoribosylformylg lycinamidine synthase	Purine metabolism
T303_01345	0.29	Phosphoribosylamino imidazole synthetase	Purine metabolism
T303_01355	0.36	Purine biosynthesis protein purH	Purine metabolism
T303_01405	0.37	Adenylosuccinate lyase	Purine metabolism
T303_08165	0.31	Ribose-phosphate pyrophosphokinase	Purine metabolism
T303_07240	0.63	Uridine/cytidine kinase	
T303_01380	0.55	Phosphoribosylamine–glycine ligase	
**Posttranslational modification, protein turnover, chaperones**			
T303_04605	0.57	Peptidase family U32	
T303_01935	0.56	Trigger factor	
T303_01640	0.47	ATP-dependent Clp protease ATP-binding protein	
T303_02240	3.24	Molecular chaperone GroES	
T303_02095	2.38	Fe–S cluster assembly protein SufB	
T303_08905	1.49	ATP-dependent Clp protease ATP-binding protein	
**Translation, ribosomal structure and biogenesis**			
T303_07405	0.69	Phenylalanyl-tRNA synthase subunit alpha	
T303_01735	0.65	30S ribosomal protein S9	
T303_09775	0.63	30S ribosomal protein S7	
T303_00530	0.63	50S ribosomal protein L15	
T303_01625	0.62	Elongation factor Ts	
T303_02860	0.62	Translation initiation factor IF-2	
T303_00580	0.61	30S ribosomal protein S17	
T303_05295	0.58	Asparaginyl-tRNA synthase	
T303_06855	0.48	Translation factor (SUA5)	
T303_01040	0.42	Tryptophanyl-tRNA synthase	
T303_06580	0.41	Peptide chain release factor 2	
T303_00615	2.97	50S ribosomal protein L23	
**Other biological processes**			
T303_03920	6.98	Pyridine nucleotide-disulfide oxidoreductase	Oxidation-reduction process
T303_07670	2.19	NAD(P)H nitroreductase	Oxidation-reduction process
T303_07355	0.35	Heme ABC transporter ATP-binding protein	Heme transport
T303_06055	0.48	PhoU family transcriptional regulator	Inorganic ion transport and metabolism
T303_05405	2.99	ATPase	Inorganic ion transport and metabolism
T303_05590	0.70	Signal recognition particle protein Srp54	
T303_02205	1.56	Preprotein translocase subunit YajC	
T303_03125	0.28	Acetyl-CoA carboxylase subunit alpha	Lipid transport and metabolism
T303_04425	0.18	Dehydrogenase	Lipid transport and metabolism
T303_03080	0.41	Acyl carrier protein	Lipid transport and metabolism
T303_08665	0.43	RNA helicase	
T303_04855	0.59	GTP-binding protein	
T303_02740	0.34	NrdR family transcriptional regulator	
T303_08865	0.49	Aminoglycoside phosphotransferase	
T303_07285	0.56	Aconitate hydratase	
T303_07275	0.63	Isocitrate dehydrogenase	
T303_01900	2.59	Hypothetical protein	
T303_04110	4.02	Branched-chain amino acid aminotransferase	
T303_08745	2.85	Copper-binding protein	
T303_04795	2.24	Cytoplasmic membrane protein	
T303_01820	0.35	Hypothetical protein	
T303_03875	2.05	Sulfurtransferase	
T303_04595	1.70	Hypothetical protein	

As shown in Table 1, we also observed that expression of several key proteins (T303_05420 and T303_05425) involved in cysteine catabolism was improved, whereas protein expression for methionine/histidine/threonine biosynthesis was repressed in ST1275 under pH 5.5 condition. It has been revealed that histidine/methionine/cysteine are essential amino acids for *Str. thermophilus* LMG18311 (Pastink et al., 2009). Thus, it is clear that cysteine metabolism plays a critical role in ST1275 under Cd2.

### Effects of pH Decrease (Cd2 vs. Cd1) on Carbohydrate Metabolism in ST1275

Lactose is the major carbon source in milk. It is observed that the LacS (T303_07870; lactose permease) was about 2-fold down regulated in ST1275 under Cd2 compared to that of Cd1 (Table 1). Our previous study showed that about 99% lactose was utilized by ST1275 after 12-h milk fermentation under Cd1, while only 24% lactose was catabolized under Cd2 at the same period of fermentation (Zisu and Shah, 2003). Thus, down-regulated LacS may be responsible for the low lactose catabolism under Cd2. Another two proteins (T303_06080 and T303_06085) were up-regulated for the metabolism of glycogen and maltodextrin which were found in our skim milk powder. We found the potential metabolism of glycogen/starch by ST1275 during our genomic study (Wu et al., 2014); however, our growth fermentation conducted for ST1275 (Supplementary Figure S1) did not show strong evidence for the metabolism of starch and maltose (key intermediate metabolite in starch metabolism) that could stimulate the growth of ST1275.

### Effects of pH Decrease (Cd2 vs. Cd1) on Other Biological Processes in ST1275

As shown in Table 1, acyl carrier protein (T303_03080) was 2.44-fold down regulated; this carrier binds all fatty acyl intermediate metabolites during fatty acid (FA) biosynthesis (Chan and Vogel, 2010). Thus, we believe that FA biosynthesis in ST1275 under Cd2 was also down-regulated due to the central role of acyl carrier protein in FA biosynthesis. Two up-regulated proteins (T303_03920, 6.98-fold; T303_07670, 2.19-fold) involved in oxidation-reduction process transforming reductive compounds to their deleterious metabolites suggesting the shift of cellular redox state in response to pH decrease. In addition, we also found five differentially expressed proteins for purine metabolism were down-regulated at least by 2-fold suggesting the nucleotide synthesis was affected by decreasing the medium pH (condition 2); most of DEPs linked to the category of translation, ribosomal structure and biogenesis were down regulated indicating potential shift of cellular translational event. It appears that pH decrease may also influence electron transfer involved in respiration process in ST1275 as evidenced by up-regulation of Fe–S cluster assembly proteins – T303_02075 and T303_02095. There are several differentially expressed proteins involved in the other processes including translation and posttranslational modifications, but we did not have a unified conclusion on these processes because both up- and down-regulated proteins were observed; several differentially expressed proteins with unknown function requires further characterization.

### DEPs From Comparisons – Cd3 vs. Cd2 (Temperature Increase) and Cd 4 vs. Cd2 (WPI Supplementation)

**FIGURE 4:**
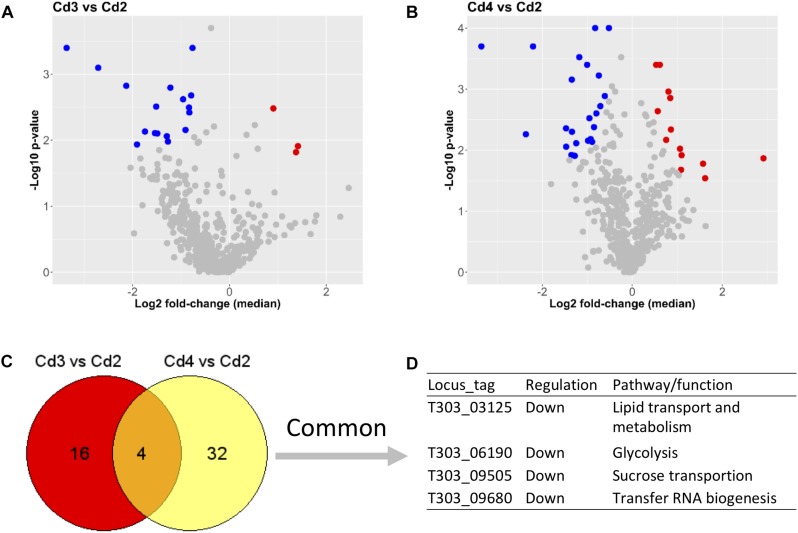
Comparisons between conditions (Cd3 and Cd4) and Cd2 based on proteome analysis. **(A)** DEPs from the comparison (Cd3 vs. Cd2). **(B)** DEPs from the comparison (Cd4 vs. Cd2); **(C,D)** Common DEPs shared by two comparisons. Dot (detected proteins) highlighted in blue indicates down-DEPs, dot (detected proteins) highlighted in red indicates up-DEPs.

Lists of DEPs from two comparisons (Cd3 vs. Cd2 and Cd4 vs. Cd2) are showed in Supplementary Table S2. As also shown in Figures 4A,B, there were only 3 down-DEPs but 17 up-DEPs in Cd3 due to temperature increase (from 37 to 40°C) when compared to Cd2, whereas 23 down-DEPs and 13 up-DEPs were found in Cd4 compared to Cd2 for assessing the effect of WPI supplementation. Obviously, there were less DEPs from above comparisons when compared to the effect of pH decrease on DEPs as shown in Figure 3. When DEPs from both comparisons were compared, only four concordant protein targets were found to be related with various different functions. This suggests that temperature increase and WPI supplementation have their own unique inducing effects on the ST175 proteome.

### Effects of Temperature Increase (Cd3 vs. Cd2) on Biological Processes in ST1275

The differentially expressed proteins from ST1275 under Cd3 (40°C) compared to that of Cd2 (37°C) are listed in Supplementary Table S2. Only three differentially up-regulated proteins (T303_02515, 2.59-fold; T303_2740, 2.66-fold; T303_04255, 1.87-fold) were detected. T303_02515 encodes a pseudouridine synthetase, one of the assembly factors, is critical for ribosome assembly in addition to two rRNAs and 33 ribosomal proteins (Jiang et al., 2007), thus ribosome assembly was believed to be improved in ST1275 under Cd3; T303_02740 is transcriptional repressor NrdR which inhibits the expression of class Ib ribonucleotide reductases under aerobic condition (Torrents et al., 2007), which is the same condition for our milk batch fermentations including Cd2 and Cd3; T303_4255 encodes a metalloprotease which may contribute to proteolysis. Other seven differentially expressed proteins (at least 2-fold down-regulation) detected belong to different biological functions. The total 8 differentially expressed proteins indicate that there were less changes in the protein expression in ST1275 under Cd2 and Cd3.

### Effects of WPI Supplementation (Cd4 vs. Cd1) on Biological Processes in ST1275

The differentially expressed proteins from ST1275 under Cd4 (WPI supplementation) compared to that of Cd2 (without WPI supplementation) is listed in Supplementary Table S2. A total of 36 differentially expressed proteins were found during the comparison between Cd4 and Cd2. Typically, the expression of acyl carrier protein (T303_03080) was improved by 7.53-fold change again under Cd4, suggesting an enhanced fatty acid biosynthesis. The up-regulation of phosphocarrier protein (T303_07270, 3.16-fold) did not provide actual evidence for the enhanced phosphoenolpyruvate-dependent sugar phosphotransferase system (PTS) since lactose is transported by LacS permease in ST1275 (Wu et al., 2014). Other differentially expressed proteins are involved in various biological processes in ST1275. Similar to the comparison of Cd3 vs. Cd2, there was very limited difference in protein expression observed under Cd4 as compared to Cd2.

### Paired Analysis of Transcriptome and Proteome Data for All Five Comparisons

**FIGURE 5:**
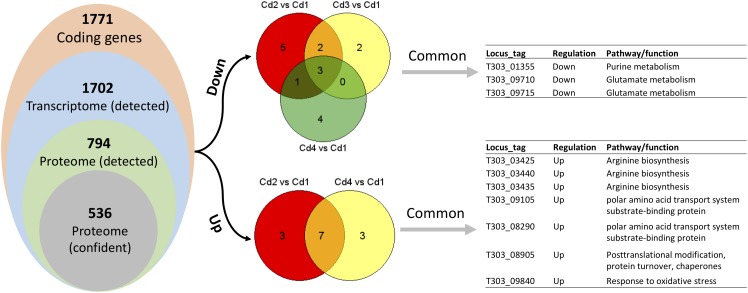
Comparisons between optimized conditions (Cd2, Cd3, and Cd4) and pH 6.5 condition (Cd1) based on paired analysis on both proteome and transcriptome data. Only DEPs and DEGs (both up and down) were used to find common differential expressed targets.

Since matched transcriptome data is available from our previous study (Wu and Shah, 2018), we performed pathway enrichment analysis with Protein Analysis Through Evolutionary Relationships (PANTHER) platform. However, using a list of DEPs for this type of analysis did not generate any significant output, thus we used a list of detected proteins (Supplementary Table S2) for enrichment analysis. As shown in Table 2, only glutamine-glutamate conversion and arginine biosynthesis were likely to be regulated concordantly at mRNA and protein level. We reasoned this phenomenon by looking at the distribution of concordant targets from both DEPs and DEGs: there were no concordant hits for two comparisons (Cd3 vs. Cd2 and Cd4 vs. Cd2) when using Cd2 as a reference; as for Cd1-referenced comparisons – 21 for Cd2 vs. Cd1, 3 for Cd3 vs. Cd1, 18 for Cd4 vs. Cd1 as indicated in Figure 5). In addition, a lot of DEPs/DEGs may not be assigned to a pathway as evidenced by only 32.69% protein-coding genes in ST1275 are connected to Kyoto Encyclopedia of Genes and Genomes (KEGG) pathways. Thus, it is not expected to have a lot of concordant pathways enriched by supplying lists of DEPs and DEGs.

**TABLE 2:** Pathway enrichment analysis with PANTHER.

**PANTHER Pathways**	**Cd2 vs. Cd1**		**Cd3 vs. Cd1**		**Cd4 vs. Cd1**	
			
	**Transcriptome DEGs**	**Proteome All detected proteins**	**Transcriptome DEGs**	**Proteome All detected proteins**	**Transcriptome DEGs**	**Proteome All detected proteins**
Arginine biosynthesis (P02728)	Up	N.S.	Up	N.S.	Up	Up
*O*-antigen biosynthesis (P02757)	N.S.	N.S.	N.S.	N.S.	N.S.	N.S.
Glutamine glutamate conversion (P02745)	Down	Down	N.S.	Down	N.S.	Down
Methionine biosynthesis (P02753)	Up	N.S.	Up	N.S.	Up	N.S.
Cysteine biosynthesis (P02737)	N.S.	Up	N.S.	N.S.	Up	N.S.
Pyruvate metabolism (P02772)	N.S.	N.S.	N.S.	N.S.	N.S.	N.S.
Chorismate biosynthesis (P02734)	N.S.	N.S.	N.S.	N.S.	N.S.	N.S.
*De novo* pyrimidine deoxyribonucleotide biosynthesis (P02739)	N.S.	Up	N.S.	N.S.	N.S.	N.S.
Histidine biosynthesis (P02747)	N.S.	Down	N.S.	Down	N.S.	N.S.
Leucine biosynthesis (P02749)	N.S.	Up	N.S.	N.S.	N.S.	N.S.
Threonine biosynthesis (P02781)	N.S.	Down	N.S.	N.S.	N.S.	N.S.
*De novo* pyrimidine ribonucleotides biosythesis (P02740)	N.S.	N.S.	N.S.	N.S.	Down	N.S.

*N.S., not significant; Up, significant (*p* < 0.05) up-regulated; Down, significant (*p* < 0.05) down-regulated.*

Later, we performed concordance analysis for DEPs and DEGs to confirm some common targets that were both regulated with the same trend. As shown in Figure 5, three concordant down-DEPs/DEGs and 7 up-DEPs/DEGs were observed from three comparisons when using Cd1 as reference control. Among those 10 concordant hits, five proteins were associated with glutamate metabolism and arginine biosynthesis; this was also demonstrated by PANTHER enrichment analysis.

## Discussion

In the present study, we performed the pairwise comparisons among the four fermentation conditions tested for ST1275 (Figures 3, 4). More specifically, when using Cd1 as the reference, we observed more DEPs of ST1275 that were consistently down-regulated under the rest three conditions, whereas less up-regulated DEPs were identified. This also suggests that the primary factor – pH decrease (Cd2) played a more important role in protein expression of ST1275 than that of second factor – either temperature increase (Cd3) or WPI supplementation (Cd4). Such observation was also evidenced by single factor-concentrated comparisons, i.e., Cd3 vs. Cd2 and Cd4 vs. Cd2. To our knowledge, this is the most comprehensive proteomic study conducted for *Str. thermophilus* in milk-based environment illustrating genome-wide protein expression in response to shifts in milk environment. However, further efforts to study different strains of *Str. thermophilus* would be able to validate the strain-specific or species-specific properties of those DEPs.

With the availability of complete genome sequence of ST1275 (Wu et al., 2014), we previously performed transcriptome study for ST1275 under four conditions tested in the present study. Among these DEGs, specifically, we noted the increased level of mRNA transcripts that are associated with EPS assembly and arginine/methionine/cysteine metabolism in pH 5.5 conditions (Cd2, Cd3, and Cd4) compared to pH 6.5 condition (Cd1) (Wu and Shah, 2018). In this proteomic study, out of 20 EPS assembly associated proteins identified in ST1275, only EpsB (T303_06415) was recognized as a down-regulated DEP. EpsB inhibits the activity of phosphorylated EpsD which is responsible for activating EpsE, the priming enzyme for synthesizing EPS repeating unit in *Str. thermophilus* [40]. Thus, down expression of EpsB might contribute to improve EPS assembly through enhanced activities of EpsE and EpsD (Minic et al., 2007; Wu and Shah, 2018). As we also observed the down transcription of *epsB* in our transcriptome study (Wu and Shah, 2018), it appears that down-regulated EpsB may contribute to EPS production under pH 5.5 conditions for this organism.

**FIGURE 6:**
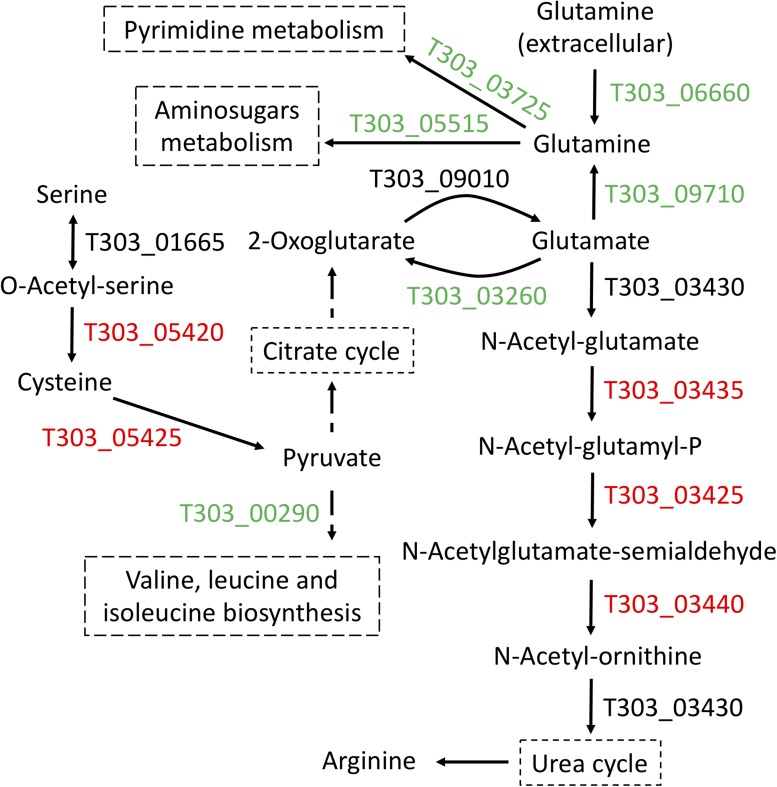
Arginine biosynthesis in ST1275 was enhanced in condition 2 as compared to that of condition 1. Gene IDs highlighted in red indicate significantly up-regulated (at least 2-fold); gene IDs (locus tag) highlighted in green indicate significantly down-regulated (at least 2-fold); gene IDs highlighted in black indicate no significantly change.

Next focus is amino acid metabolism, we performed three comparisons (Cd2 vs. Cd1, Cd3 vs. Cd1, and Cd4 vs. Cd1) to understand the concordant targets of DEPs induced by pH decrease (Figure 3). This analysis provided confidence to confirm glutamate metabolism was down regulated under pH decrease condition (Figure 3E), as evidenced by PATHER enrichment analysis (Table 2); more importantly, concordance analysis of DEPs and DEGs from paired transcriptome and proteome data has also indicated down expression of glutamate metabolism and up-regulation of arginine biosynthesis in ST1275 under pH 5.5 conditions (Figure 5). Further pathway analysis for those DEPs demonstrated a clear link for the discordance between glutamate catabolism and arginine biosynthesis (Figure 6) in ST1275. It appears that more glutamate could be utilized for arginine biosynthesis under pH 5.5 conditions. Arginine biosynthesis in *Str. thermophilus* LMG 18311 was enhanced during co-culturing with *Lb. bulgaricus* (Herve-Jimenez et al., 2009). Another study showed improved growth of *Str. thermophilus* T1C2 by arginine supplementation through arginine decarboxylation event for maintaining intracellular pH homeostasis (Huang et al., 2016); however, the arginine decarboxylase pathway is absent in most of the completely sequenced *Str. thermophilus* strains including several commercial strains ST1275, CNRZ1066, LMG18311, LMD-9, ND03 and MN-ZLW-002. Also, supplementation of glutamate to the chemically defined medium showed a slight growth increase for *Str. thermophilus* (Pastink et al., 2009). Thus, the actual functions of elevated arginine biosynthesis are still not clear for *Str. thermophilus*. One limitation of this study is the absence of amino acid profiles for these fermented milks. Further experimental validation whether EpsB and arginine biosynthesis for ST1275 are associated with enhanced EPS is necessary.

In this study, we tested effects of pH decrease, temperature increase and WPI supplementation on protein expression profiles in ST1275. Due to detection limit by MS technology, only the abundantly expressed proteins in ST1275 under the four conditions examined were observed. The pH decrease had great influence on protein expression of ST1275 including stress responses to fermentation conditions, glutamate catabolism, arginine biosynthesis, cysteine catabolism, lactose uptake and fatty acid biosynthesis. Through paired analysis of multi-omics data and concordance analyses based on shared proteins, we were able to confidently conclude that glutamate-arginine metabolism was one of key cellular changes in ST1275 under pH 5.5 conditions and EpsB might be a potential target for manipulating EPS biosynthesis in ST1275. One limitation of this study is the presence of non-catabolizable maltodextrin and gluco-oligosaccharide in the fortified milk base; this challenged the technical measurement of EPSs under such conditions since the repeating units of polysaccharides and EPSs could be overlapped, thus the size exclusion would be not accurate for purifying EPSs of ST1275. However, we believe our multi-omics approach could still be valuable for understanding physiological responses of ST1275 in response to fermentation conditions. Further investigations on the association between EPS production and critical pathways such as arginine biosynthesis and stress responses will be necessary for understanding the enhanced EPS production machinery in ST1275 under pH 5.5 condition.

## Data Availability

Analysis results of proteomic data are included as Supplementary Tables S1, S2. Transcriptomic data are available from the Sequence Read Archive (SRA) repository of National Center for Biotechnology Information (accession number: PRJNA395470): 8 libraries (matched samples used in this study: Cd1_rep2, Cd1_rep3, Cd2_rep1, Cd2_rep3, Cd3_rep2, Cd3_rep3, Cd4_rep2, and Cd4_rep3) out of 12 total libraries (triplicates for each condition in our previous transcriptome study) were used in this study as paired transcriptome analysis for iTRAQ-based proteomic analysis. The iTRAQ-based proteomic data was deposited to ProteomeXchange respiratory (accession ID: PXD013699) through MassIVE submission portal.

## Author Contributions

NS and QW designed the study. QW and AP conducted the milk fermentation and protein extraction. QW and HC performed the iTRAQ-labeling, peptide fractioning and cleaning, and proteomic data acquisition and analysis. QW analyzed the transcriptomic data, interpreted the multi-omics data, and drafted the manuscript and substantively revised it. NS reviewed and edited the manuscript.

## Conflict of Interest Statement

The authors declare that the research was conducted in the absence of any commercial or financial relationships that could be construed as a potential conflict of interest.

## Supplementary Material

FIGURE S1 Growth curves of ST1275 in M17 medium supplemented with or without individual lactose, maltose, and starch. Lactose was supplemented at 20 g/L (separate autoclave); maltose was also supplemented at 20 g/L (separate autoclave); water soluble starch was supplemented at 1 g/L (pasteurization). OD600 values of cultures of each condition were measured in a 96-well plates by loading 200 μL of cultures to each well.

FIGURE S2 Correlation analysis for 536 detected protein-coding genes by both transcriptome (log2 fold-change) and proteome (log2 fold-change-median) results for all comparisons.

TABLE S1 List of RNA-seq analysis results including DEGs from comparisons between two selected conditions.

TABLE S2 List of proteomic analysis results including DEPs from comparisons between two selected conditions.
