# Supplementary figures and images for: Functional Genomic Analyses of Exopolysaccharide-Producing Streptococcus thermophilus ASCC 1275 in Response to Milk Fermentation Conditions
Source: Front Microbiol. 2019 Aug 23;10:1975. doi: 10.3389/fmicb.2019.01975 (PMC6716118; doi:10.3389/fmicb.2019.01975)

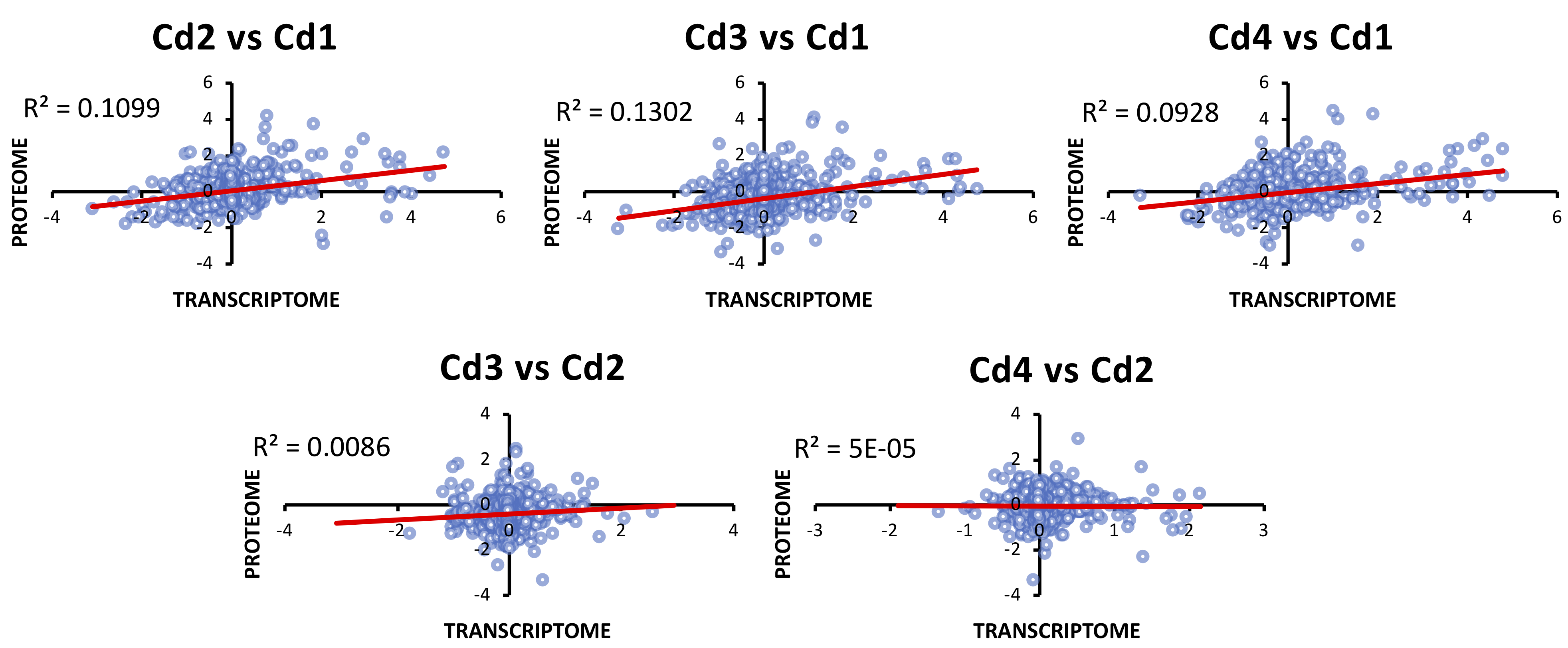

Supplement: FIGURE S2 — Correlation analysis for 536 detected protein-coding genes by both transcriptome (log2 fold-change) and proteome (log2 fold-change-median) results for all comparisons. [file Image_2.TIFF]
